# Integrating RNA-Seq, Transcription Factor Annotation, and WGCNA Identifies Key Candidate Genes in Upland Cotton Seedlings Under Short-Term Drought Stress

**DOI:** 10.3390/genes17091068

**Published:** 2026-09-03

**Authors:** Gang Wang, Wanli Han, Zhibin Zhang, Xiaomei Ma, Hai Zhao, Yandi Yao, Fuxiang Zhao, Jinxin Qiao, Xiang Zhang, Yu Yu, Hongguang Liu

**Affiliations:** 1 College of Water Conservancy and Architectural Engineering, Shihezi University, Shihezi 832000, China; wg80669@163.com; 2 Cotton Research Institute, Xinjiang Academy of Agricultural and Reclamation Science, Shihezi 832000, China; 3 Institute of Cotton Research, Chinese Academy of Agricultural Sciences, Anyang 455000, China; 4 Xinjiang Shihezi Academy of Agricultural Sciences, Shihezi 832000, China

**Keywords:** upland cotton, drought tolerance, RNA-seq, WGCNA, candidate genes

## Abstract

**Background:** Drought is one of the major abiotic stresses that affect and limit cotton growth and production. However, transcriptome differences between drought-tolerant and drought-susceptible cotton lines remain largely unknown. **Methods and Results:** In this study, two upland cotton cultivars, the drought-tolerant XLZ80 and drought-sensitive XLZ61, were subjected to comparative phenotypic and transcriptomic analyses under drought stress. Phenotypic evaluation showed that XLZ80 exhibited only mild leaf wilting, whereas XLZ61 displayed severe wilting symptoms after drought stress. RNA-seq analysis revealed that differentially expressed genes in XLZ80 were specifically enriched in pathways related to phosphatidylinositol signaling, phenylalanine metabolism, MAPK signaling, and betaine biosynthesis, while DEGs in XLZ61 were primarily involved in basal metabolic processes. A total of 9302 core DEGs were identified across and between the cultivars and were grouped into eight dynamic expression clusters containing 841 transcription factors. Weighted gene co-expression network analysis further identified three key modules associated with drought tolerance. Twelve hub genes, including *GH_D02G2153* (MADS-box) and *GH_A05G1087* (bZIP), were identified as central regulators. qRT-PCR validation confirmed that these genes exhibited faster and stronger induction in the tolerant cultivar. In summary, this study deepens the transcriptional-level understanding of drought stress responses in cotton and provides valuable gene resources for breeding drought-resistant cultivars.

## 1. Introduction

Crops are consistently challenged by a range of environmental stresses, including extreme temperatures, drought, pests, and diseases [1]. Among these, drought stands out as a major abiotic stressor that severely limits plant growth and crop productivity. Climate change has further exacerbated this threat by increasing the frequency and severity of drought events worldwide [1,2,3]. Cotton is an important crop used worldwide to produce natural fibers, edible oils, and plant protein [4]. However, it is particularly susceptible to the significant effects of drought stress during the seedling stage [5]. Drought stress typically induces premature flowering, boll shedding, and substantial yield and fiber quality losses in cotton [6]. Although traditional breeding has achieved significant progress in improving yield and fiber quality, enhancing drought tolerance has received comparatively less attention. Therefore, developing drought-resistant cotton cultivars is imperative for ensuring sustainable cotton production in water-limited environments.

RNA sequencing has emerged as a powerful tool for dissecting the transcriptional regulatory networks underlying plant drought responses [7]. For example, integrated transcriptome and metabolome analyses revealed that flavonoid and jasmonic acid pathways are essential for alkali stress responses in *Gossypium hirsutum* [8]. RNA-seq analyses have revealed that drought stress triggers the up-regulation of genes involved in osmotic adjustment, antioxidant defense, and hormone signaling while down-regulating those associated with cell expansion and photosynthesis in cotton [9]. RNA-seq identified key NAC transcription factors in rice, such as *ONAC129* [10] and *ONAC45* [11], that confer drought and heat tolerance. Transcriptome profiling identified key transcription factors in soybean such as *GmbZIP1* [12] and *GmWRKY27* [13] that enhance drought tolerance by regulating stomatal closure and root architecture. Multi-genotype RNA-seq studies in cotton have highlighted the importance of *GhPP2C1* [14] and *GhSnRK2* [15] in mediating drought signal transduction. Similarly, transcriptome profiling in maize uncovered *ZmZIM23* as a major drought-responsive gene regulating water-use efficiency [16]. Co-expression analyses across multiple cotton genotypes further identified *GhDREB2A-A* and *GhHSFA6B-D* as crucial regulators enhancing yield under water deficit via ABA-independent pathways [17]. These studies demonstrate the efficacy of transcriptomic approaches in elucidating drought-response mechanisms and identifying candidate genes for crop improvement.

Building on these advances, we conducted a multi-timepoint transcriptome analysis on young root tips of the drought-tolerant cultivar XLZ80 and drought-sensitive cultivar XLZ61 at 0, 2, 4, and 6 h after drought stress to capture the dynamic transcriptional landscape of drought tolerance in upland cotton at the seedling stage. Subsequently, differentially expressed genes (DEGs) were systematically analyzed through co-expression clustering, GO and KEGG enrichment, transcription factor annotation, and weighted gene co-expression network analysis (WGCNA). Key candidates were further validated by qRT-PCR. This integrated approach identified core gene modules central to the drought response, providing new insights into the molecular basis of drought tolerance and delivering valuable gene resources for the molecular breeding of drought-resistant cotton varieties.

## 2. Materials and Methods

### 2.1. Plant Materials, Growth Conditions and Drought Treatment

The experimental materials used in this study were the upland cotton varieties XLZ80 (drought-tolerant) and XLZ61 (drought-sensitive), both provided by the Cotton Research Institute, Xinjiang Academy of Agricultural Sciences (Xinjiang, China). Plump, uniformly sized seeds were selected and sown in seedling cups filled with a mixture of substrate and vermiculite in a 1:2 volume ratio. After emergence, seedlings with uniform growth were carefully washed to remove residual substrate and transferred to a hydroponic system. The seedlings were grown at 28 °C/25 °C (day/night) under a 16 h light/8 h dark photoperiod. At the three-leaf stage, drought stress was simulated by adding 15% (*w*/*v*) polyethylene glycol (PEG6000) to the nutrient solution. Samples were collected at 0, 2, 4, and 6 h after PEG6000 treatment, immediately frozen in liquid nitrogen, and stored at −80 °C for subsequent RNA-seq and qRT-PCR analyses.

### 2.2. Total RNA Extraction and RNA-Seq

Total RNA was extracted from approximately 100 mg of each sample using the Polysaccharide Polyphenol Plant Total RNA Extraction Kit (Tiangen, Beijing, China), following the manufacturer’s protocol. RNA purity and concentration were determined using a NanoDrop spectrophotometer (Thermo Fisher Scientific, Waltham, MA, USA), and RNA integrity was assessed using an Agilent 2100 Bioanalyzer (Agilent Technologies, Santa Clara, CA, USA). Only samples with high-quality RNA (RIN ≥ 7.0) were used for library preparation and sequencing, which was conducted by Biomarker Technologies Co., Ltd. (Beijing, China), on the Illumina HiSeq 2500 platform.

### 2.3. Sequencing Data Filtering, Assembly and Differential Gene Expression Analysis

Raw reads were processed with Fastp to remove adaptor sequences, reads containing >5% unknown bases (N), and low-quality reads [18]. The resulting clean reads were then aligned to the *G. hirsutum* reference genome (ZJU-AD1_v2.1) [19] using HISAT2 [20], followed by transcript assembly with StringTie [21] and expression quantification in FPKM (Fragments Per Kilobase of transcript per Million mapped reads). Differential expression analysis was performed using DESeq2 [22], with genes showing FDR < 0.01 and |log_2_(Fold Change)| > 1 defined as DEGs. Functional enrichment analysis of DEGs was carried out with the R package clusterProfiler4 [23] for Gene Ontology (GO) and Kyoto Encyclopedia of Genes and Genomes (KEGG) annotation, while transcription factors (TFs) were predicted and classified using the Plant Transcription Factor Database (PlantTFDB) [24].

### 2.4. Construction of Gene Co-Expression Network

Co-expression network construction was performed using the WGCNA package in R [25]. A soft-threshold power (*β*) of eight was selected to achieve scale-free topology. The automatic network construction function blockwiseModules was used to identify gene modules with parameters minModuleSize = 30 and mergeCutHeight = 0.25. Modules with eigengene correlation above 0.75 were merged. To identify the key hub genes related to cotton’s drought resistance, this study, based on the key drought-resistant co-expression modules, calculated the module characteristic gene correlation (kME) values of the genes within each module and defined the top 4 genes with the highest kME values in each module as the hub genes of that module. Network visualization was performed using Cytoscape 3.10.4 automation [26].

### 2.5. qRT-PCR Analysis

Gene-specific primers were designed using Primer 5.0 based on target cDNA sequences (Table S1). Total RNA was extracted using the RNAprep Pure Polysaccharide Polyphenol Plant Total RNA Kit (Tiangen, Beijing, China), and three biological replicates were analyzed per sample. RNA concentration and purity were determined using a NanoDrop 2000 spectrophotometer (Thermo Fisher Scientific, Waltham, MA, USA). cDNA was synthesized using the M-MLV RTase cDNA Synthesis Kit (TaKaRa, Kusatsu, Shiga, Japan). Quantitative real-time PCR (qRT-PCR) was performed using iTaq Universal SYBR Green Supermix (Bio-Rad, Hercules, CA, USA) on a Bio-Rad CFX96 Real-Time PCR system. Relative expression levels were calculated using the 2^−ΔΔCt^ method [27], with *GhUBQ7* as the internal reference gene. The data were analyzed using IBM SPSS 27.0 software, with *p* < 0.01 and *p* < 0.05 used as the criteria for statistical significance.

## 3. Results

### 3.1. The Upland Cotton Xlz80 Is More Drought-Tolerant than Xlz61 by Phenotypic Analysis

**Figure 1:**
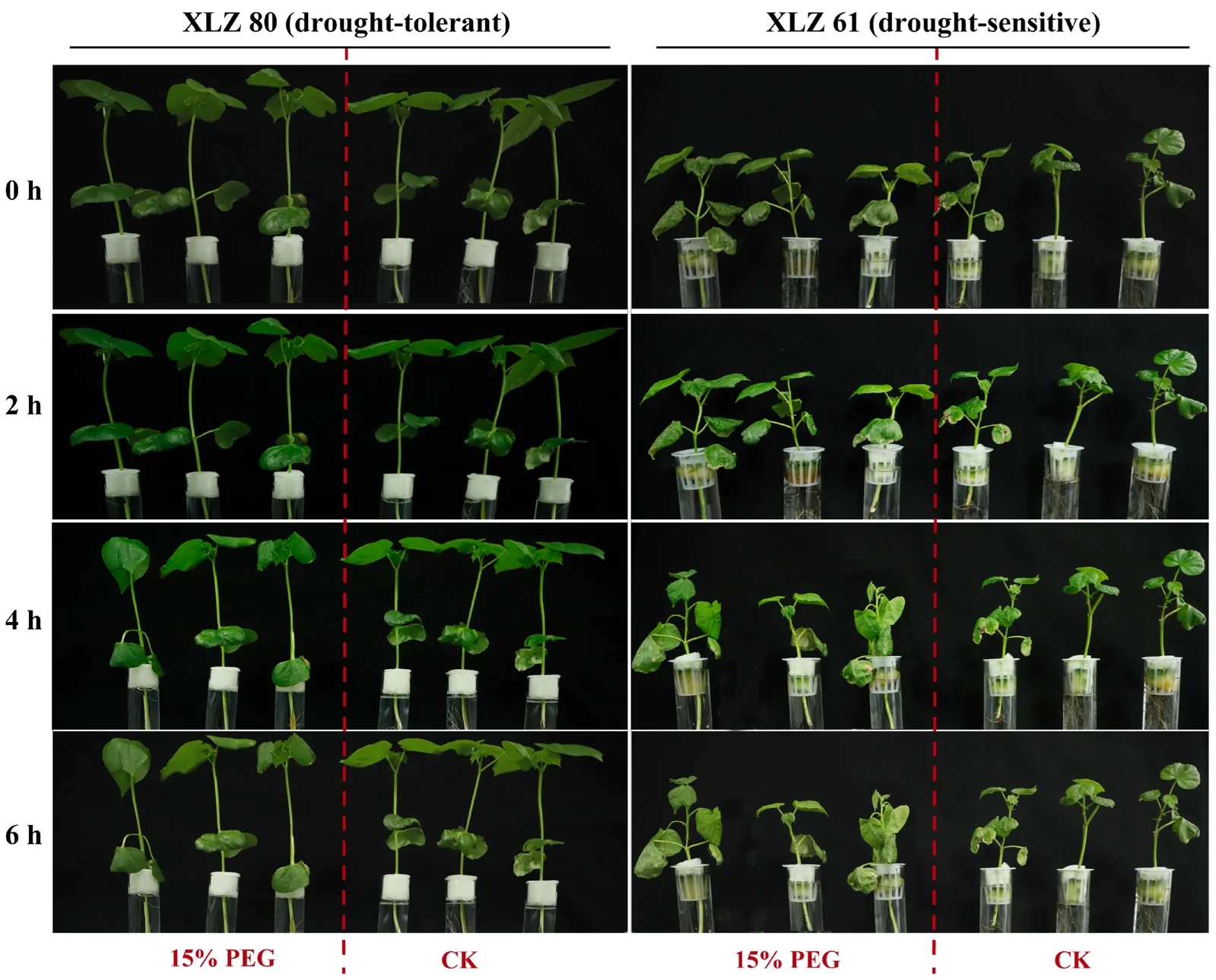
Phenotypes of upland cotton seedlings under PEG stress.

To assess drought tolerance in the *G. hirsutum* cultivars XLZ80 and XLZ61, we simulated drought with 15% (*w*/*v*) PEG 6000 and monitored phenotypic changes under stress (Figure 1). After 2 h of treatment, the leaves of XLZ80 showed virtually no visible change, whereas XLZ61 exhibited slight wilting. At 4 h, XLZ80 exhibited mild wilting, whereas XLZ61 showed more severe leaf dehydration. After 6 h, XLZ80 maintained moderate wilting, whereas XLZ61 showed further deterioration.

### 3.2. The Reliability of the RNA-Seq Data Was Confirmed Through Correlation and PCA Analysis

A total of 351.31 Gb of high-quality clean data was generated from three biological replicates at each time point. For all samples, the proportion of Q30 bases was more than 91.24%, and the GC content was more than 44.00% (Table S2). To evaluate experimental reproducibility, Pearson correlation coefficients were calculated from FPKM values; biological replicates within the same treatment showed coefficients greater than 0.91 (Figure S1a). Principal component analysis (PCA) further demonstrated tight clustering of replicates and clear separation between the cultivars (Figure S1b), indicating that genotype was the primary source of variation. In addition, qRT-PCR validation of ten randomly selected genes (three technical replicates) revealed expression trends highly consistent with the RNA-seq data (Figure S2, r = 0.87, *p* < 0.01). Collectively, these results indicated that the RNA-seq dataset was of high quality and exhibited strong reproducibility, supporting subsequent differential expression and functional analyses.

### 3.3. Characterization of Differentially Expressed Genes in Xlz61 and Xlz80 During Drought Response via Time-Series Transcriptomic Analysis

**Figure 2:**
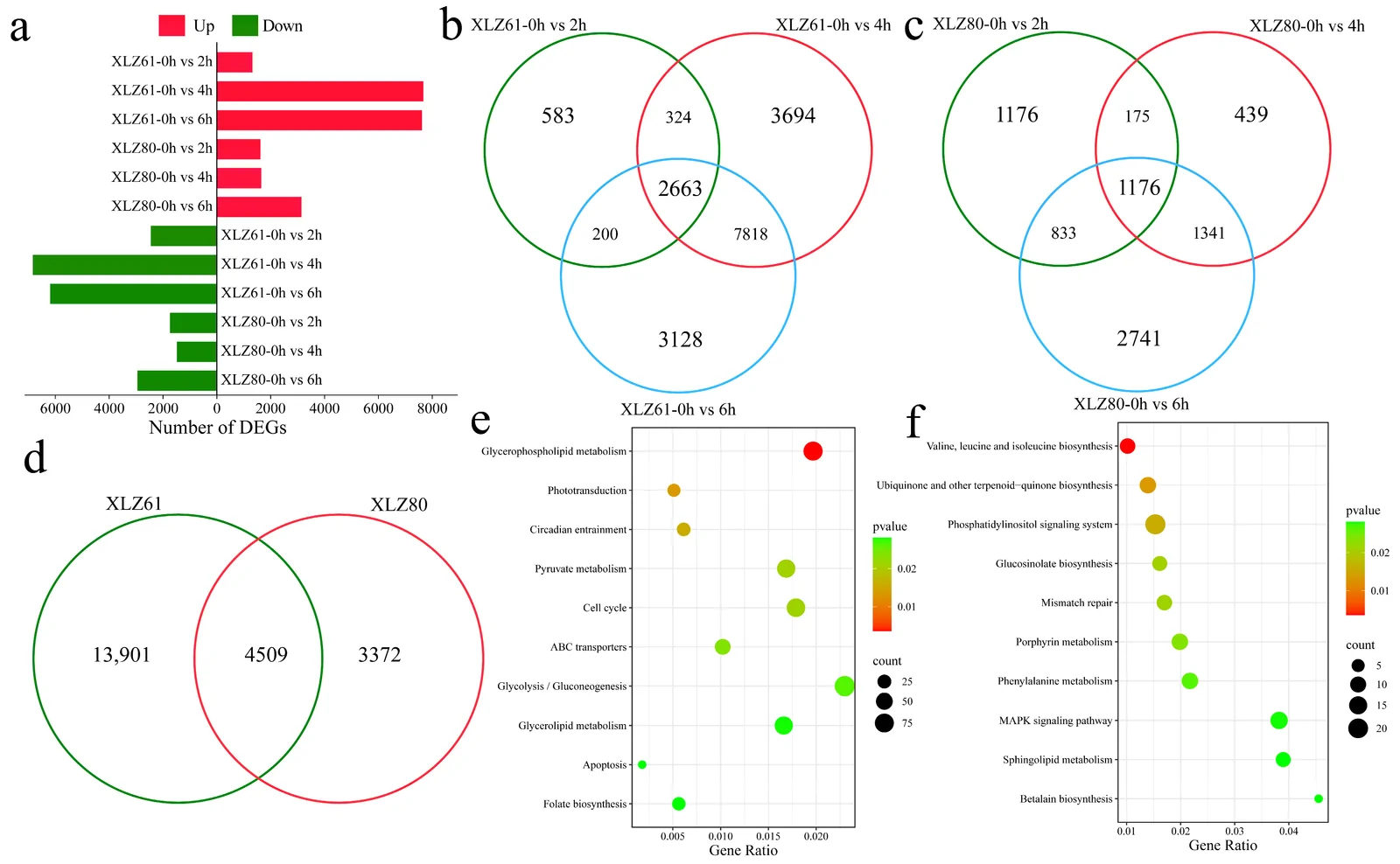
Analysis of drought-responsive differential gene expression in XLZ80 and XLZ61. (**a**) The numbers of up- and down-regulated DEGs. Venn diagrams display time-point-specific and shared DEGs in (**b**) XLZ61 and (**c**) XLZ80. (**d**) A Venn diagram identifying DEGs shared between or specific to XLZ80 and XLZ61. KEGG pathway enrichment analysis of the (**e**) XLZ61-specific and (**f**) XLZ80-specific DEGs.

Differential expression analysis was performed by comparing each drought treatment (2 h, 4 h, 6 h) with the control (0 h) for both cultivars. In XLZ61, a total of 18,410 DEGs were detected, of which 2663 were shared across the time points (Figure 2a,b). Over time, 3770, 14,499, and 13,809 DEGs were identified at 2, 4, and 6 h, respectively. Of these, 1319 were up-regulated and 2451 down-regulated at 2 h, with 583 DEGs unique to this time point; 7665 were up-regulated and 6834 down-regulated at 4 h, with 3694 time-point-specific DEGs; and 7617 were up-regulated and 6192 down-regulated at 6 h, with 3128 time-point-specific DEGs. In XLZ80, 7881 DEGs were identified in total, including 1176 shared across the time points (Figure 2a,c). At 2, 4, and 6 h, 3360, 3131, and 6091 DEGs were detected, respectively. Specifically, 1618 were up-regulated and 1802 down-regulated at 2 h, with 1176 DEGs unique to this time point; 1646 were up-regulated and 1485 down-regulated at 4 h, with 439 time-point-specific DEGs; and 3144 were up-regulated and 2947 down-regulated at 6 h, with 2801 time-point-specific DEGs. Between-cultivar comparisons revealed 21,782 DEGs in total, of which 13,901 were unique to XLZ61, 3372 were unique to XLZ80, and 4509 were shared by both cultivars (Figure 2d). KEGG enrichment analysis of the 13,901 XLZ61-specific DEGs indicated significant enrichment in glycerophospholipid metabolism, phototransduction, circadian entrainment, pyruvate metabolism, cell cycle, ABC transporters, glycolysis/gluconeogenesis, glycolipid metabolism, apoptosis, and folate biosynthesis (Figure 2e). In contrast, the 3372 XLZ80-specific DEGs were significantly enriched in valine, leucine and isoleucine biosynthesis; ubiquinone and other terpenoid-quinone biosynthesis; phosphatidylinositol signaling system; glucosinolate biosynthesis; mismatch repair; porphyrin metabolism; phenylalanine metabolism; MAPK signaling pathway; sphingolipid metabolism; and betalain biosynthesis (Figure 2f).

### 3.4. Differentially Expressed Genes Between Xlz80 and Xlz61 Increased Significantly as Drought Persisted

**Figure 3:**
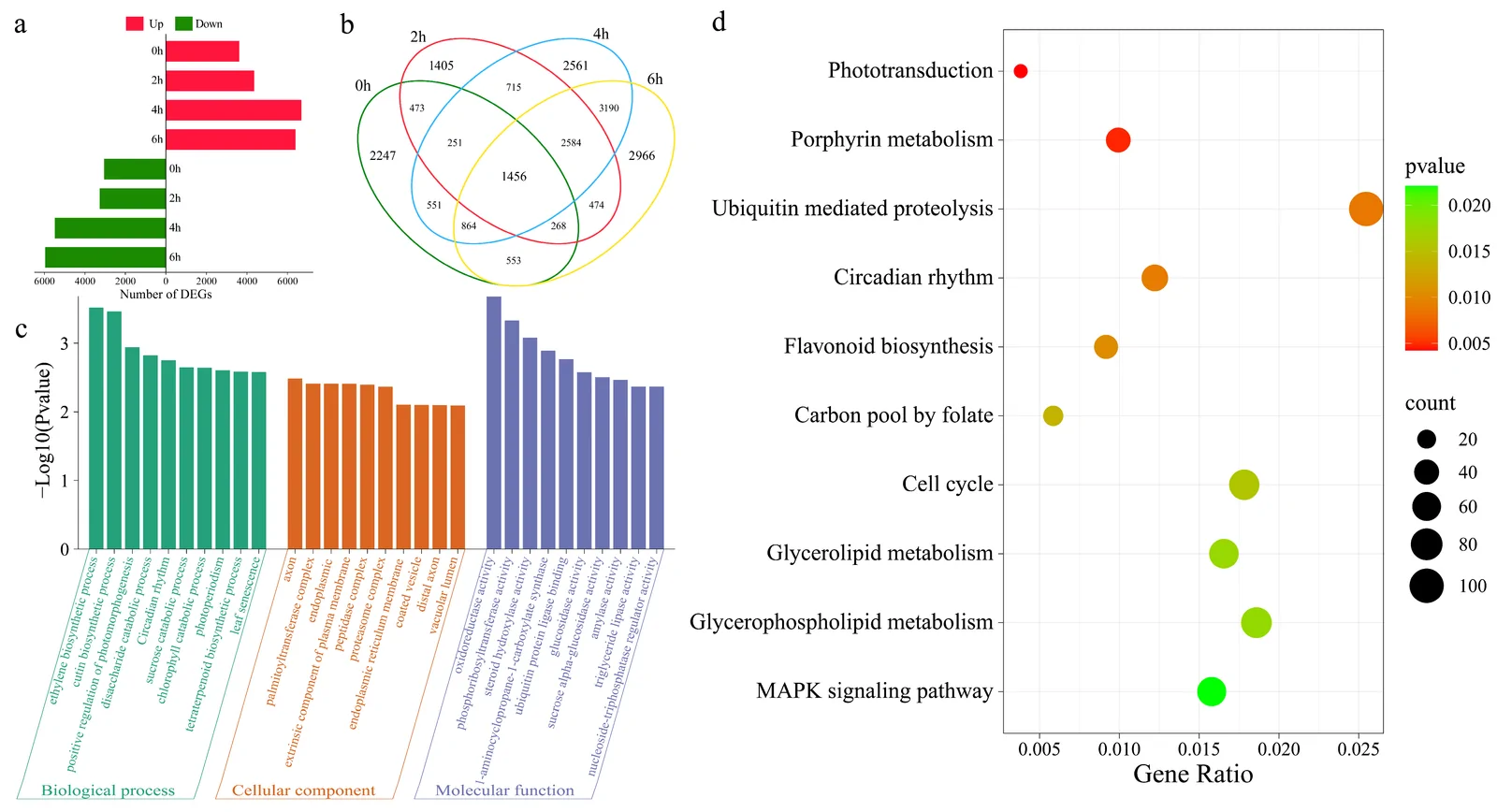
Identification and functional characterization of cultivar-specific DEGs. (**a**) Numbers of the up- and down-regulated DEGs. (**b**) A Venn diagram of cultivar-specific and shared DEGs. (**c**) Gene Ontology (GO) and (**d**) KEGG pathway enrichment analysis of the DEGs.

A total of 13,895 DEGs were identified between XLZ80 and XLZ61 under the same stress duration, with 1456 shared across all time points (Figure 3a,b). At 0 h, 6663 DEGs were detected (3619 up-regulated and 3044 down-regulated), including 2247 specific to this point. At 2 h, 7626 DEGs were detected (4358 up and 3268 down), with 1405 time-point-specific DEGs. At 4 h, 12,172 DEGs were detected (6687 up and 5485 down), with 2561 specific DEGs. At 6 h, 12,355 DEGs were detected (6394 up and 5961 down), with 2966 specific DEGs. Functional enrichment of the 13,895 between-cultivar DEGs revealed significant overrepresentation in the GO biological processes ethylene biosynthetic process, cutin biosynthetic process, positive regulation of photomorphogenesis, disaccharide catabolic process, circadian rhythm, sucrose catabolic process, chlorophyll catabolic process, photoperiodism, tetraterpenoid biosynthetic process, and leaf senescence (Figure 3c). KEGG pathway enrichment was significant for phototransduction, porphyrin metabolism, ubiquitin-mediated proteolysis, circadian rhythm, flavonoid biosynthesis, one-carbon pool by folate, cell cycle, glycerolipid metabolism, glycerophospholipid metabolism, and MAPK signaling pathway (Figure 3d).

### 3.5. Clustering of 9302 Overlapping Genes Reveals Distinct Drought-Response Patterns Between the Two Varieties

**Figure 4:**
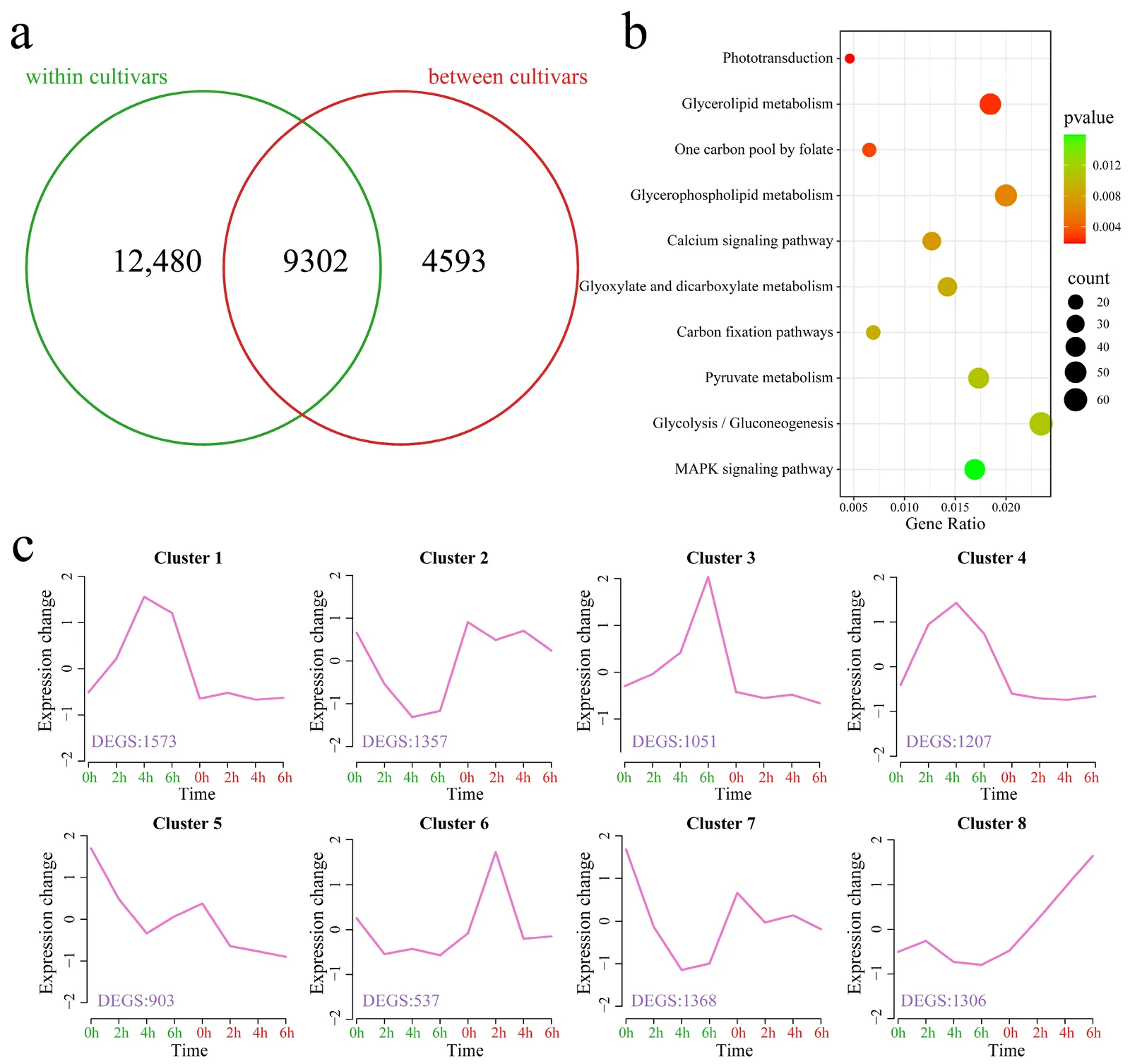
Shared and cultivar-specific drought-responsive DEGs in XLZ80 and XLZ61. (**a**) A Venn diagram of DEGs across comparisons. (**b**) KEGG enrichment analysis of shared DEGs. (**c**) Expression clustering of shared DEGs with cluster sizes indicated (green: XLZ61, red: XLZ80).

To further pinpoint key genes conferring drought tolerance, we intersected DEGs identified within the cultivars and between the cultivars, yielding 9302 DEGs present in both contrasts; subsequent analyses focused on these 9302 DEGs (Figure 4a). KEGG enrichment showed significant annotation of these genes in phototransduction, glycerolipid metabolism, one-carbon pool by folate, glycerophospholipid metabolism, calcium signaling pathway, glyoxylate and dicarboxylate metabolism, carbon fixation pathways, pyruvate metabolism, glycolysis/gluconeogenesis, and the MAPK signaling pathway (Figure 4b). Based on expression pattern clustering, the 9302 DEGs were partitioned into eight clusters with dynamic expression characteristics (Figure 4c). Cluster 1 (1573 genes) increased after drought in XLZ61 but remained essentially stable in XLZ80. Cluster 2 (1357 genes) decreased after stress in XLZ61, with no obvious change in XLZ80. Cluster 3 (1051 genes) rose at 6 h in XLZ61 but showed a slight decrease in XLZ80. Cluster 4 (1207 genes) peaked at 4 h in XLZ61, while remaining broadly stable in XLZ80. Cluster 5 (903 genes) declined in both cultivars. Cluster 6 (537 genes) decreased continuously in XLZ61 but transiently increased at 2 h and then declined in XLZ80. Cluster 7 (1368 genes) decreased in both cultivars, with a more pronounced decline in XLZ61. Cluster 8 (1306 genes) decreased slightly in XLZ61 but increased steadily in XLZ80, reaching a maximum at 6 h.

### 3.6. Members of 11 Transcription Factor Families Exhibit Variety-Dependent Regulatory Patterns Under Drought Stress

**Figure 5:**
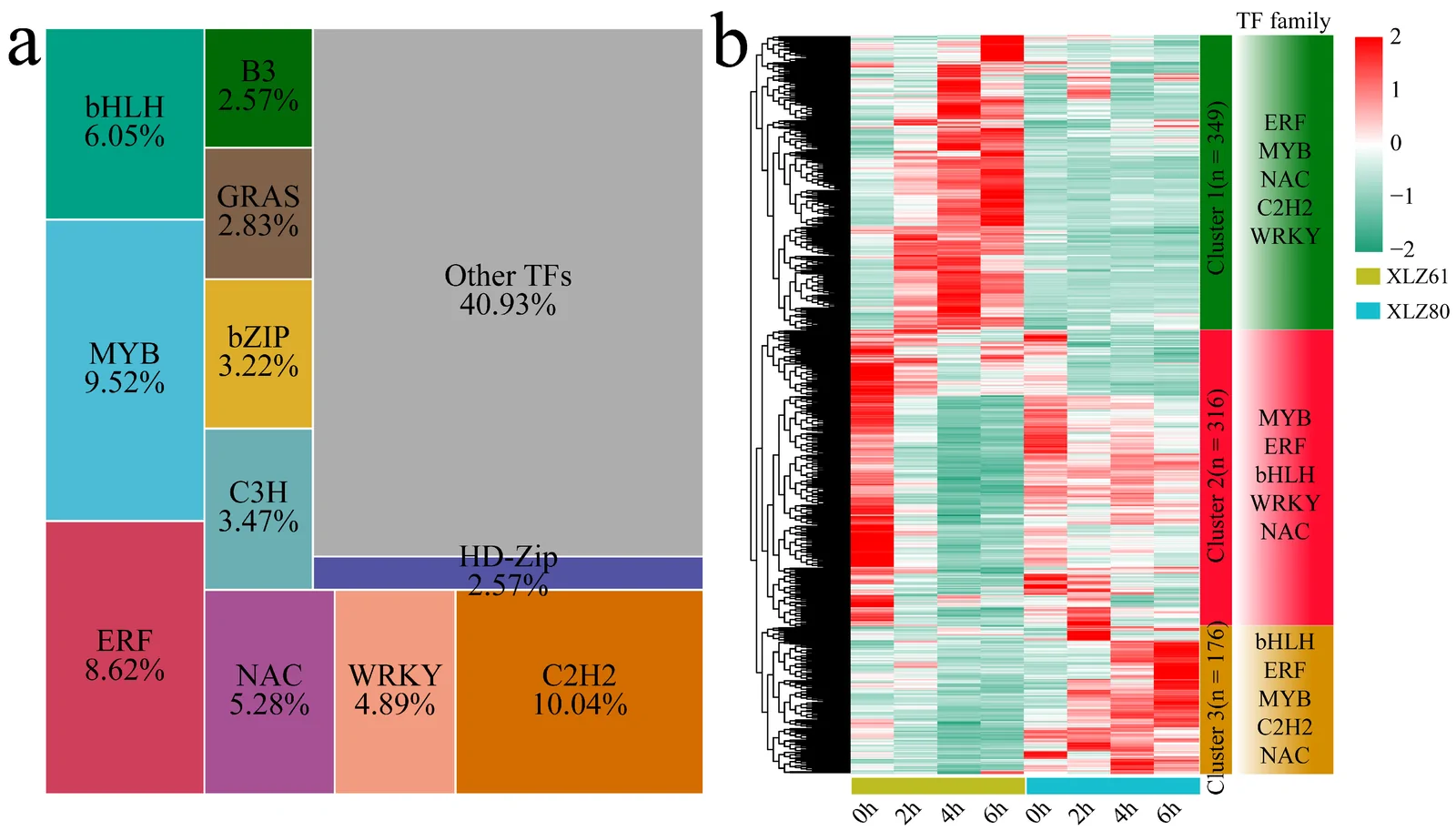
Characterization of drought-responsive transcription factors. (**a**) Composition of key differentially expressed TF families. (**b**) Clustering and expression patterns of TFs across cultivars and time points.

Transcription factors play key regulatory roles in plant growth, development, and responses to abiotic stress. Among the 9302 DEGs, we identified 841 differentially expressed transcription factors (TFs). The most represented families—constituting the major responders—included ERF, MYB, bHLH, NAC, WRKY, C2H2, C3H, bZIP, GRAS, B3, and HD-Zip (Figure 5a). Expression pattern clustering grouped these 841 TFs into three clusters with distinct dynamics (Figure 5b). Cluster 1 (349 TFs) showed markedly increased expression after drought in XLZ61, while remaining essentially stable in XLZ80; this cluster was enriched for ERF, MYB, NAC, C2H2, and WRKY members. Cluster 2 (316 TFs) exhibited a decreasing trend upon stress in both cultivars, comprising primarily the MYB, ERF, bHLH, WRKY, and NAC families. Cluster 3 (176 TFs) showed no notable change in XLZ61 but was clearly up-regulated in XLZ80 and mainly included the bHLH, ERF, MYB, C2H2 and NAC families.

### 3.7. Twelve Core Drought-Tolerance Candidate Genes Were Identified Through Analysis of Gene Co-Expression Networks

**Figure 6:**
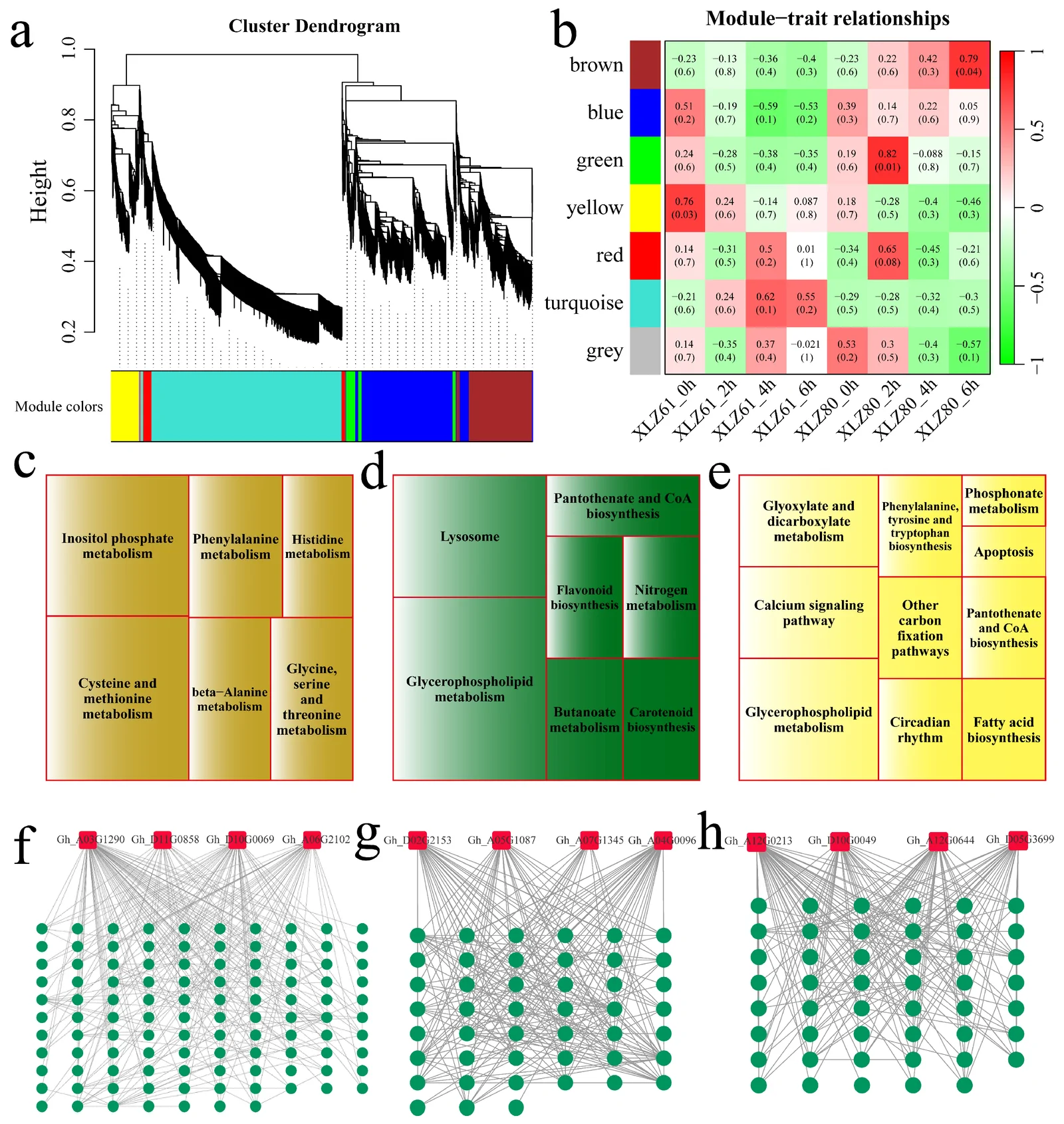
WGCNA of drought-stressed transcriptome. (**a**) Gene clustering dendrogram and module assignment. (**b**) Correlation heatmap of module eigengenes with experimental conditions. (**c**–**e**) KEGG enrichment in key modules: brown (**c**); green (**d**); yellow (**e**). (**f**–**h**) Co-expression networks for brown (**f**), green (**g**), and yellow (**h**) modules.

To dissect the gene regulatory architecture underlying drought responses in cotton and to mine core drought-tolerance genes, we performed WGCNA on the expression matrix of 9302 DEGs. A scale-free co-expression network was constructed, and genes with highly correlated expression profiles were grouped into modules using the dynamic tree-cutting algorithm, with modules denoted by distinct colors. In total, seven co-expression modules were identified (Figure 6a). To pinpoint modules most closely associated with drought-tolerance phenotypes, we correlated module eigengenes with cultivar/time point combinations for XLZ80 and XLZ61. The brown module was significantly associated with XLZ80 at 6 h of drought, the green module with XLZ80 at 2 h, and the yellow module with XLZ61 at 0 h (Figure 6b). These associations suggest that the brown and green modules are enriched for key genes that positively contribute to the drought tolerance of XLZ80. KEGG pathway enrichment of genes within these key modules revealed biologically coherent functions. Genes in the brown module were enriched in histidine metabolism, β-alanine metabolism, inositol phosphate metabolism, cysteine and methionine metabolism, phenylalanine metabolism, and glycine, serine and threonine metabolism (Figure 6c). The green module was enriched in glycerophospholipid metabolism, butanoate metabolism, lysosome, carotenoid biosynthesis, nitrogen metabolism, pantothenate and CoA biosynthesis, and flavonoid biosynthesis. The yellow module was enriched in calcium signaling pathway; glycerophospholipid metabolism; other carbon fixation pathways; glyoxylate and dicarboxylate metabolism; apoptosis; pantothenate and CoA biosynthesis; phenylalanine, tyrosine and tryptophan biosynthesis; phosphonate metabolism; circadian rhythm; and fatty acid biosynthesis. These pathways align well with established drought-response mechanisms in plants, supporting the functional relevance of the identified modules.

We next examined intramodular connectivity by correlating each gene with its module eigengene to assess its network centrality. By selecting highly connected genes, four hub genes were identified per key module. Functional annotations were inferred from Arabidopsis homologs (Table S3). *GH_A03G1290* encodes an NAD (*p*)-linked oxidoreductase involved in the oxidation–reduction process. *GH_D11G0858* encodes a YABBY transcription factor implicated in the regulation of leaf development. *GH_D10G0069* encodes cinnamyl alcohol dehydrogenase participating in phenylpropanoid biosynthesis. *GH_A06G2102* encodes a C2H2 transcription factor involved in the abscisic-acid-activated signaling pathway. *GH_D02G2153* encodes a MADS-box transcription factor associated with responses to environmental stresses. *GH_A05G1087* encodes a bZIP transcription factor associated with responses to environmental stresses. *GH_A07G1345* encodes a calcium-dependent protein kinase involved in the response to stimulus. *GH_A04G0096* encodes glycerophosphoryl diester phosphodiesterase in glycerophospholipid metabolism. *GH_A12G0213* encodes a UDP-glucosyltransferase involved in the biosynthesis of plant secondary metabolites. *GH_D10G0049* encodes chitinase associated with glutathione metabolism. *GH_A12G0644* encodes pantetheine phosphate adenylyltransferase in pantothenate and CoA biosynthesis. *GH_D05G3699* encodes ascorbate oxidase in ascorbate and aldarate metabolism. These hub genes occupy central positions within their respective modules and likely serve as key regulatory nodes in the drought-response network.

### 3.8. In the Drought-Tolerant Variety Xlz80, Core Candidate Genes Are Induced Earlier and Exhibit a Stronger Response, as Demonstrated by qRT-PCR Experiments

**Figure 7:**
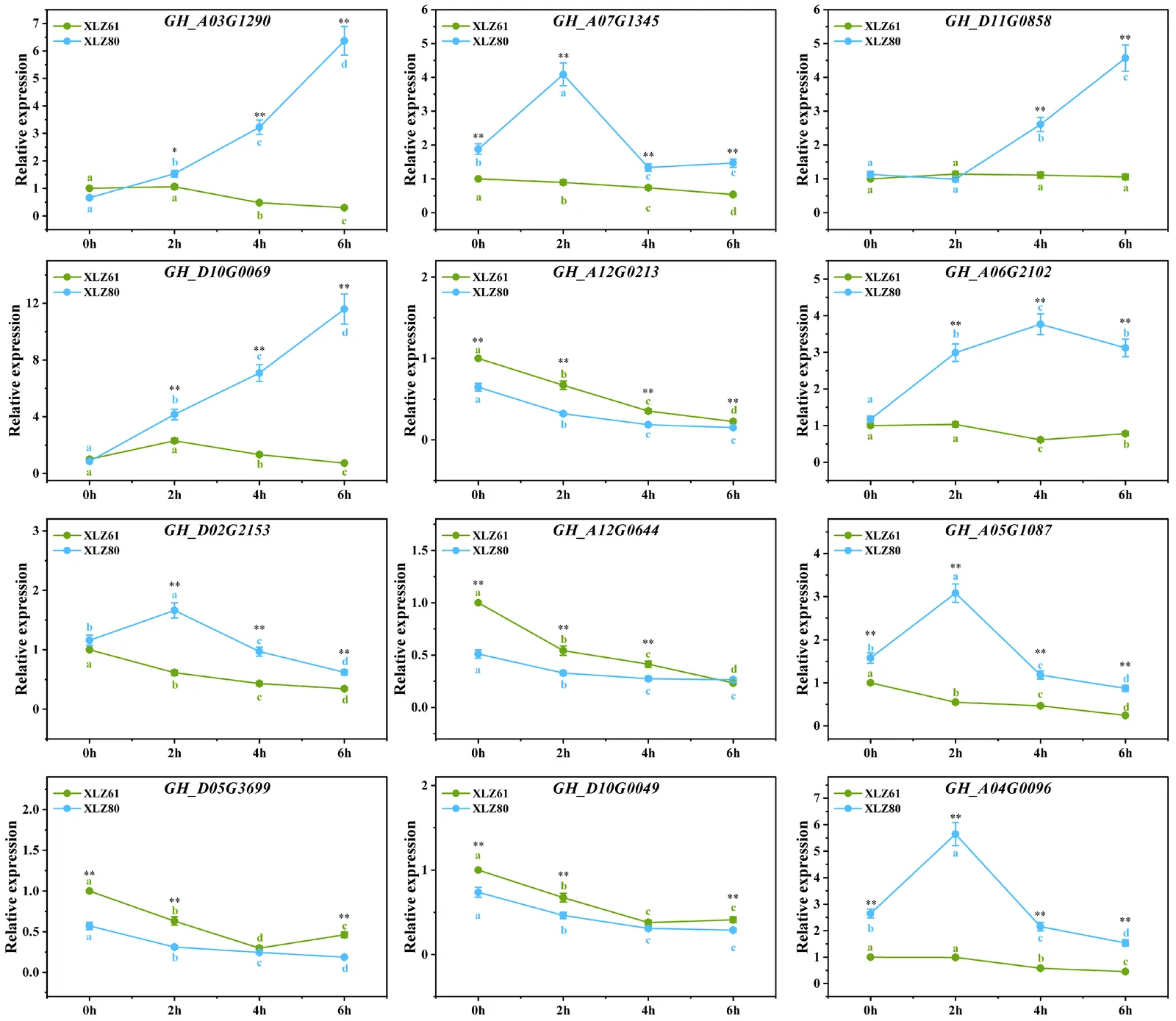
Validation of candidate genes by qRT-PCR. Expression levels of 12 randomly selected candidate genes. At the same stage, an independent-sample *t*-test was used to compare the expression differences in the candidate genes between the two cotton varieties. The significance level is indicated by asterisks (* *p* < 0.05, ** *p* < 0.01); within the same cotton variety, for expression quantity differences in the candidate genes in different drought stress stages, one-way ANOVA combined with the least significant difference method and Duncan’s multiple comparison method was used for statistical analysis, and group differences are indicated by letter notation.

To validate the reliability of the candidate genes and elucidate their dynamic expression during the drought response, we performed qRT-PCR on the 12 prioritized genes (Figure 7). The expression profiles clearly segregated into distinct patterns. Notably, most genes exhibited faster and stronger induction in the drought-tolerant cultivar XLZ80. For example, the transcription factor genes *GH_D11G0858* (YABBY), *GH_A06G2102* (C2H2), *GH_D02G2153* (MADS) and *GH_A05G1087* (bZIP) were significantly up-regulated as early as 2 h after stress in XLZ80, whereas in the sensitive cultivar XLZ61 their induction was delayed or even repressed. Moreover, although several genes were induced in both cultivars, the magnitude of induction was consistently higher in XLZ80, suggesting a more efficient regulatory capacity for drought defense. In summary, RNA-seq-guided identification and qRT-PCR validation revealed a suite of key genes and signaling pathways underlying drought tolerance in cotton.

## 4. Discussion

Drought is a major abiotic constraint that severely affects cotton growth, yield, and fiber quality worldwide [28]. Although plants have evolved diverse strategies to mitigate water deficit, the molecular basis underlying drought tolerance in cotton remains incompletely understood. In this study, we simulated drought conditions using 15% PEG6000 and compared the responses of two *G. hirsutum* cultivars with contrasting drought tolerance, XLZ80 and XLZ61, through phenotypic evaluation and transcriptome profiling across multiple time points. Consistently with previous knowledge that drought tolerance relies on rapid stomatal regulation, osmotic adjustment, and maintenance of cellular homeostasis [29], XLZ80 exhibited stronger adaptive capacity, whereas XLZ61 showed pronounced stress symptoms. Transcriptomic analyses revealed that XLZ61 primarily activated pathways associated with basal metabolic maintenance, phototransduction, and cell-cycle progression, which may support short-term acclimation but appear insufficient for sustained defense. As drought persisted, the inadequate early response in XLZ61 led to metabolic imbalance, ROS accumulation, membrane lipid peroxidation, and ultimately irreversible cellular damage, manifested by chloroplast breakdown and photosynthetic collapse. These findings identify key drought-responsive pathways and candidate genes that contribute to tolerance differences and highlight potential targets for enhancing cotton performance under water-limited conditions.

RNA-seq analysis revealed extensive transcriptional reprogramming under drought conditions. Notably, the number of DEGs was much higher in XLZ61 than in XLZ80, consistent with the tendency of sensitive genotypes to mount more drastic, yet potentially less beneficial, transcriptional responses [30]. In contrast, pathways such as the MAPK cascade, calcium signaling, and phototransduction were significantly enriched in XLZ80. MAPK cascades are central to drought signal perception and transduction, activating downstream transcription factors to regulate tolerance genes. In cotton, signaling via the *GhMAP3K62*-*GhMKK16*-*GhMPK32* pathway can phosphorylate *GhEDT1*, thereby inducing the ABA biosynthetic gene *GhNCED3*, promoting ABA accumulation and enhancing drought tolerance [31]. Similarly, the *GhMPK7*-*GhSDIRIP1* module has been reported to improve drought tolerance by positively regulating ABA signaling [32]. In our data, MAPK-related genes displayed a more stable up-regulation pattern in the tolerant XLZ80 but fluctuated in XLZ61, suggesting a homeostatic regulatory mechanism associated with drought tolerance.

In all, 841 differentially expressed TFs were identified, largely from key families including ERF, MYB, NAC, WRKY, and bHLH, which play central roles in cotton’s drought response. For example, *GhNAC4* enhances drought adaptation by regulating secondary cell-wall biosynthesis genes (*GhNST1*) and ribosomal protein genes (*GhRPL12* and *GhRPL18p*), thereby promoting secondary wall deposition in roots and maintaining ribosomal protein homeostasis [33]. Expression pattern analyses indicated that many TFs were highly expressed in the sensitive XLZ61 or were down-regulated in both cultivars, implying that XLZ61 relies on a rapid but potentially unstable stress response, whereas the tolerant XLZ80 achieves more durable adaptation through gradual tuning. In particular, differential expression of bHLH members underscores the multilayered nature of transcriptional control under drought. In Cluster 3, XLZ80 maintained a more stable TF expression program, which may help preserve transcriptional homeostasis and support long-term drought responses.

Given the thousands of DEGs, prioritizing functionally important genes is challenging. WGCNA effectively addressed this by constructing a drought-response co-expression network and identifying hub genes within the XLZ80-associated brown and green modules. These hubs are concentrated in pathways known to mediate drought responses, such as glycerophospholipid metabolism, calcium signaling, and phenylpropanoid metabolism. Genes including *Gh_A06G2102* (C2H2) and *Gh_A07G1345* (a calcium-dependent protein kinase) occupy central positions in the network, suggesting pivotal roles in drought signal perception and downstream responses. C2H2 TFs are well documented in ABA signaling [34], while CDPKs are key nodes in drought signal transduction [35]. These results provide clear targets for enhancing cotton drought tolerance via gene editing or molecular breeding. Furthermore, existing studies have shown that the physical location clustering of co-regulated genes (i.e., their clustered distribution on the chromosomes) can also serve as a potential mechanism for transcriptional coordination [36]. This is different from the clustering analysis based on expression profiles in this study, but the two have high complementary significance.

Despite these advances, limitations remain. Our work focused primarily on seedling-stage roots under acute drought stress. However, mechanisms may differ across developmental stages and tissues under prolonged drought. Moreover, while we validated the expression patterns of candidate genes via qRT-PCR, this validation was confined to transcriptional correlation and did not establish a causal relationship between these genes and drought tolerance. Functional characterization through the generation of overexpression and mutant lines is essential to definitively establish the roles of these candidate genes in drought regulation. Therefore, future studies should generate overexpression and mutant lines of these candidate genes in upland cotton to validate their functions under both short-term and long-term drought stress conditions. Furthermore, our comparative analysis was restricted to only two cultivars, which limits the assessment of genetic diversity and the generalizability of our findings. Therefore, future studies should screen additional drought-tolerant and drought-sensitive cultivars to dissect the expression patterns of these candidate genes across broader genetic backgrounds. Our findings should be interpreted as a prioritized list of high-confidence candidate genes, rather than a functional characterization of drought regulatory mechanisms. Future studies should therefore pursue dynamic, multi-stage, multi-organ analyses to establish a comprehensive regulatory atlas of cotton drought tolerance, and integrate functional genetic studies with epigenomic and metabolomic layers to further refine the drought-response network.

## 5. Conclusions

By integrating seedling-stage drought phenotyping with RNA-seq, we found that the tolerant cultivar XLZ80 exhibited only mild morphological damage under drought stress and displayed a transcriptional response distinct from that of the sensitive cultivar XLZ61, driven primarily by genotype-specific regulatory mechanisms. Functional enrichment indicated that DEGs in XLZ80 were concentrated in the phosphatidylinositol signaling system, the MAPK signaling pathway, and secondary metabolism, whereas XLZ61 was biased toward regulation of basal metabolic processes. From this together with WGCNA, we identified three core modules closely associated with drought tolerance and prioritized 12 hub genes. This study provides valuable gene resources and a theoretical basis for dissecting the molecular mechanisms of drought tolerance in cotton and establishes a solid foundation for enhancing drought resilience through genetic engineering and marker-assisted breeding.

## Data Availability

Raw data of the transcriptome analyzed in this work are available at the NCBI Sequence Read Archive (https://www.ncbi.nlm.nih.gov/sra, accessed on 10 January 2026) with the accession number PRJNA1375438.

## Supplementary Materials

The following supporting information can be downloaded at: https://www.mdpi.com/article/10.3390/genes17091068/s1, Figure S1: Assessment of transcriptome relationships under progressive drought in cultivars XLZ80 and XLZ61; Figure S2: Verify the RNA-seq data through qRT-PCR analysis; Table S1: All primers used in this study; Table S2: Summary statistics for RNA-seq analysis of *G. hirsutum* samples under drought; Table S3: Candidate gene functional annotation.
