# Supplementary material for: Integrating RNA-Seq, Transcription Factor Annotation, and WGCNA Identifies Key Candidate Genes in Upland Cotton Seedlings Under Short-Term Drought Stress
Source: Genes (Basel). 2026 Sep 3;17(9):1068. doi: 10.3390/genes17091068 (PMC13606565; doi:10.3390/genes17091068)
Supplement: Supplementary file 1 [file genes-17-01068-s001.zip › Fig S1. Assessment of transcriptome relationships under progressive drought in cultivars XLZ80 and XLZ61.pdf]

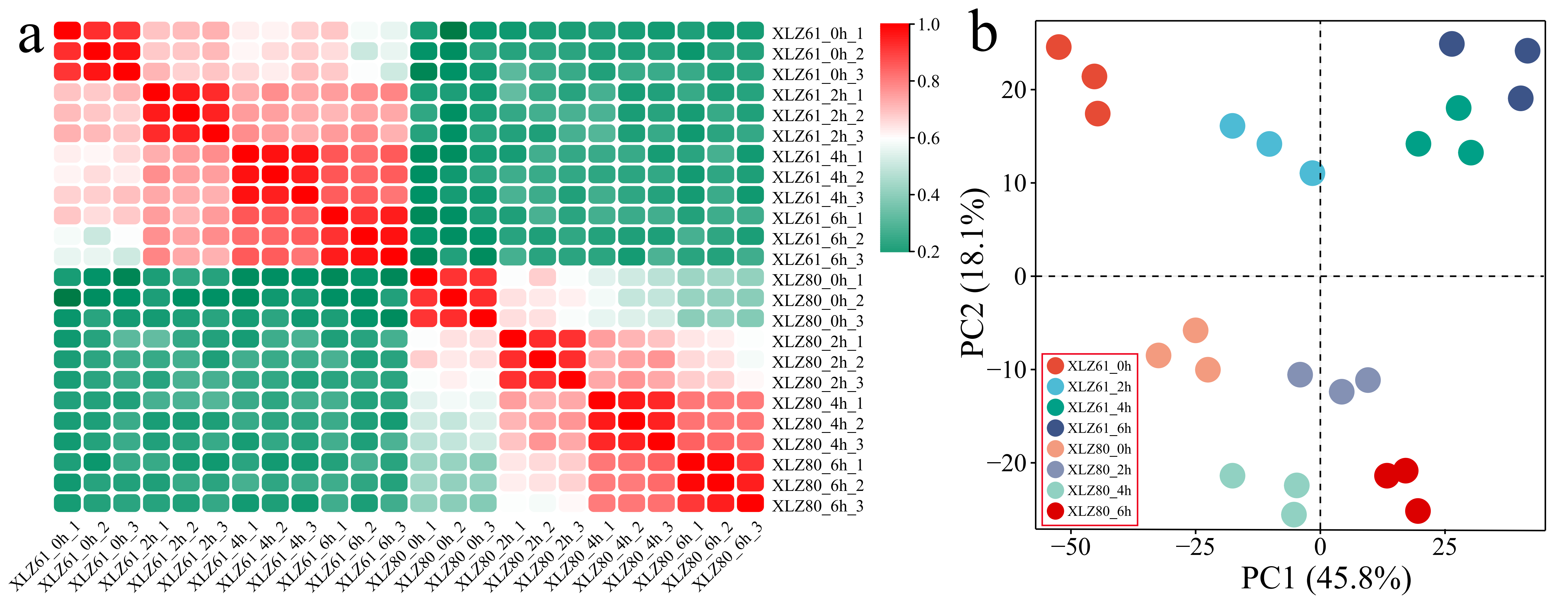
a
b
XLZ61_0h_1
XLZ61_0h_2
XLZ61_0h_3
XLZ61_2h_1
XLZ61_2h_2
XLZ61_2h_3
XLZ61_4h_1
XLZ61_4h_2
XLZ61_4h_3
XLZ61_6h_1
XLZ61_6h_2
XLZ61_6h_3
XLZ80_0h_1
XLZ80_0h_2
XLZ80_0h_3
XLZ80_2h_1
XLZ80_2h_2
XLZ80_2h_3
XLZ80_4h_1
XLZ80_4h_2
XLZ80_4h_3
XLZ80_6h_1
XLZ80_6h_2
XLZ80_6h_3
1.0
0.8
0.6
0.4
0.2
PC1 (45.8%)
PC2 (18.1%)
−50
−25
0
25
20
10
0
−10
−20
XLZ61_0h
XLZ61_2h
XLZ61_4h
XLZ61_6h
XLZ80_0h
XLZ80_2h
XLZ80_4h
XLZ80_6h
