# Supplementary material for: Integrating RNA-Seq, Transcription Factor Annotation, and WGCNA Identifies Key Candidate Genes in Upland Cotton Seedlings Under Short-Term Drought Stress
Source: Genes (Basel). 2026 Sep 3;17(9):1068. doi: 10.3390/genes17091068 (PMC13606565; doi:10.3390/genes17091068)
Supplement: Supplementary file 1 [file genes-17-01068-s001.zip › Fig S2. Verify the RNA-seq data through qRT-PCR analysis; Table S1 All primers used in this study.pdf]

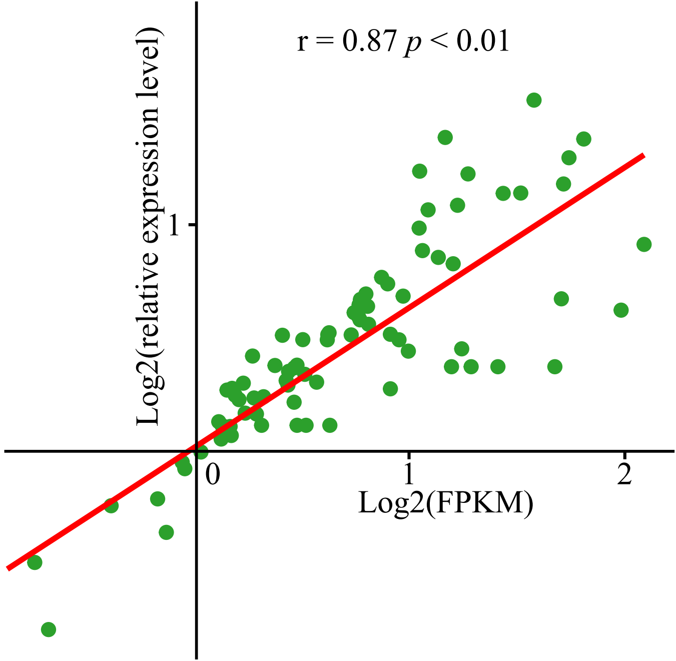

r = 0.87 p < 0.01
Log2(relative expression level)
1
0
1
2
Log2(FPKM)
